# Mitochondrial Glutathione in Diabetic Nephropathy

**DOI:** 10.3390/jcm4071428

**Published:** 2015-07-09

**Authors:** Lawrence H. Lash

**Affiliations:** Department of Pharmacology, Wayne State University School of Medicine, 540 East Canfield Avenue, Detroit, MI 48201, USA; E-Mail: l.h.lash@wayne.edu; Tel.: +1-313-577-0475; Fax: +1-313-577-6739

**Keywords:** diabetic nephropathy, mitochondria, oxidative stress, glutathione, transport, gene expression

## Abstract

Although there are many etiologies for diabetic nephropathy (DN), one common characteristic of all cases involves mitochondrial oxidative stress and consequent bioenergetic dysfunction. As the predominant low-molecular-weight, intramitochondrial thiol reductant, the mitochondrial glutathione (mtGSH) pool plays important roles in how this organelle adapts to the chronic hyperglycemia and redox imbalances associated with DN. This review will summarize information about the processes by which this important GSH pool is regulated and how manipulation of these processes can affect mitochondrial and cellular function in the renal proximal tubule. Mitochondria in renal proximal tubular (PT) cells do not appear to synthesize GSH *de novo* but obtain it by transport from the cytoplasm. Two inner membrane organic anion carriers, the dicarboxylate carrier (DIC; *Slc25a10*) and 2-oxoglutarate carrier (OGC; *Slc25a11*) are responsible for this transport. Genetic modulation of DIC or OGC expression *in vitro* in PT cells from diabetic rats can alter mitochondrial function and susceptibility of renal PT cells to oxidants, with overexpression leading to reversion of bioenergetic conditions to a non-diabetic state and protection of cells from injury. These findings support the mtGSH carriers as potential therapeutic targets to correct the underlying metabolic disturbance in DN.

## 1. Introduction

Nephropathy is a common complication of both Type 1 and Type 2 diabetes and is a frequent cause of death in diabetic patients [1]. The diabetic kidney is characterized by increased perfusion, which generates increased glomerular filtration and intraglomerular pressure [2]. Pathological changes result in initial microalbuminuria, which progresses to more extensive proteinuria, loss of tubular filtration, and ultimately renal failure. As many as 40% of diabetic patients eventually develop clinically manifest diabetic nephropathy (DN) [3,4,5] and diabetes is the most common cause of end-stage renal disease in the U.S. [6]. The prevalence of diabetes in the U.S. and other Western countries indicates that prevention and treatment of diabetic renal disease is a critical public health issue [2].

One strategy to develop an effective therapeutic approach to treat DN is to target elements of the fundamental, underlying cause of the disease. Despite glycemic control being a critical element in treating or preventing renal disease in diabetes [2,7], it is still important to treat the underlying biochemical mechanism of renal cellular damage. Various manifestations of oxidative and nitrosative stress are believed to be the underlying mechanism by which chronic hyperglycemia causes renal cellular damage [8,9,10,11,12,13,14]. In particular, decreases in renal glutathione (GSH) levels have been observed [9,10,11,13,15] and dietary supplementation with GSH can provide some degree of protection against many of the pathologies associated with DN [16]. Renal cellular responses to the diabetic state are more complex than depletion or oxidation of antioxidants such as GSH, however, in that compensatory up-regulation of several antioxidant enzymes has also been observed [17]. Furthermore, there are some discrepancies in the literature about the occurrence and exact molecular nature of the redox changes in the kidneys and other tissues [18]. Nonetheless, it is clear that redox status, in particular that of the GSH system, plays a critical, underlying role in the pathophysiology of DN.

**Figure 1:**
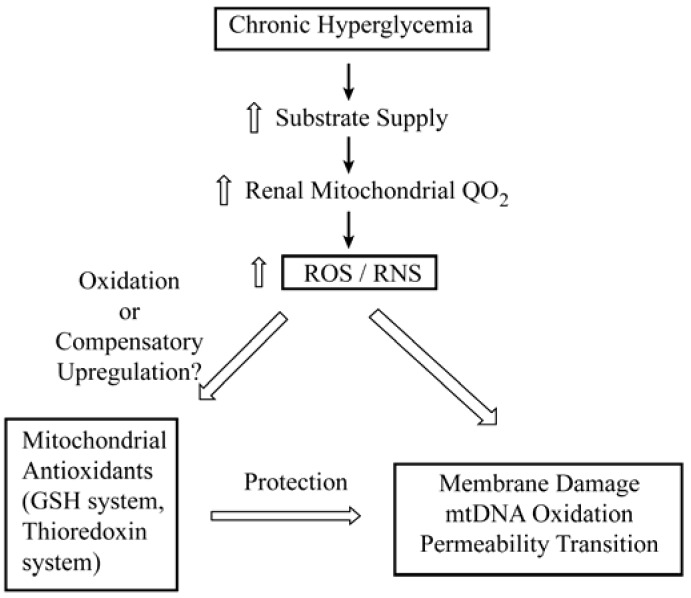
General scheme of major events leading from chronic hyperglycemia to mitochondrial dysfunction. Abbreviations: GSH, glutathione; RNS, reactive nitrogen species; ROS, reactive oxygen species.

Besides changes in redox status, changes in cellular energetics are also a fundamental response associated with DN. Moreover, the concept has developed in recent years of mitochondrial dysfunction, including alterations in mitochondrial redox status, playing a fundamental role in a broad array of diseases, pathological states, and degenerative processes [19]. Hence, metabolic disturbances due to mitochondrial dysfunction have been associated not only with DN [20], but also cancer, neurodegenerative and neuromuscular diseases, and aging. Due to the critical, underlying role of both mitochondrial dysfunction and oxidative stress and the predominance of the GSH redox system in mitochondria, it would seem logical to suggest that the mtGSH pool may be a good choice as a therapeutic target. A summary scheme of the impact of chronic hyperglycemia on renal mitochondria is illustrated in Figure 1. The scheme illustrates how elevated glucose levels lead to increased flux of carbohydrate substrates into the mitochondria, which results in increased respiration. The increased oxygen consumption rate is in turn associated with increased production and release of reactive oxygen species (ROS), such as superoxide anion and hydroxyl radical, and reactive nitrogen species (RNS), in particular peroxynitrite, which can result in damage to mitochondrial membranes and DNA if not countered by the function of antioxidants. As discussed below, the antioxidants (primarily the GSH and thioredoxin systems) may either be oxidized by the ROS and RNS or may undergo compensatory upregulation, depending on various factors such as exposure conditions and bioenergetic status.

To develop potential treatments that involve some manipulation of mtGSH, it is of course necessary to understand how this GSH pool is regulated. Hence, this brief review will first discuss discovery of the mechanism by which the mtGSH pool is derived, including the rationale and experimental steps used to establish the mechanism. Studies on the mtGSH pool in DN and a related renal pathological state, namely compensatory renal hypertrophy after uninephrectomy, will then be reviewed to illustrate how the pathways by which mtGSH are altered. Inasmuch as the altered bioenergetic and redox state of the renal proximal tubule influences how these cells handle and respond to drugs and toxic chemicals, studies on the impact of changes in mitochondrial function and mtGSH status on these processes in rat kidney and a rat renal PT cell line will be reviewed. Finally, preliminary studies conducted to apply these principles to development of a therapeutic approach to treat the mitochondrial dysfunction and oxidative stress associated with DN and other chronic diseases will be discussed.

## 2. Determination of mtGSH Status: Transport *versus* Synthesis and Identification of Carriers

### 2.1. Background and Identification of mtGSH Carriers in Renal Mitochondria

The history of biochemical and toxicological studies on the mtGSH pool in liver and kidney were summarized in several review articles over the past decade [21,22,23,24,25] and will not be extensively discussed here. The main point is that there has been significant interest in mtGSH for understanding mechanisms of cellular function and response to toxicants for more than 50 years. Despite this, the concept that the mtGSH pool in tissues such as kidney and liver represents a distinctly regulated compartment of this important thiol did not arise until the 1980s and that GSH was transported into mitochondria was not considered until the early 1990s. Schnellmann [26] was the first to demonstrate carrier-mediated transport of GSH into suspensions of isolated renal cortical mitochondria from rats. His studies showed differences in transport activity under different respiratory states but did not identify a specific mechanism of transport or the carrier(s) involved.

Earlier studies in rat liver mitochondria [27] and subsequent studies in rat kidney mitochondria [28] could find no evidence of *de novo* synthesis of GSH within mitochondria, implying that the mtGSH pool in the matrix had to derive from the cytoplasm by transport across the mitochondrial outer and inner membranes. Based on the net negative charge of GSH at physiological pH and that the mitochondrial matrix is negatively charged relative to the cytoplasm, movement of GSH from the cytoplasm into the mitochondrial matrix is energetically unfavored. Consequently, any transport of GSH into the mitochondria must be coupled to some input of energy. Typical energy inputs for such transport processes include coupling to the mitochondrial membrane potential and counter-transport or co-transport with another ion or metabolite. Because there are four potential charged groups on the GSH molecule (three negative and one positive), and considering the typically neutral-to-slightly acidic pH of the cytoplasm (pH = 7.0) and modestly alkaline pH of the mitochondrial matrix (pH = 7.8), the net charge on GSH molecules should vary between −1 and −2.

Taking the anionic nature of the GSH molecule into account, the initial hypothesis that we proposed was that GSH was transported into renal and hepatic mitochondria by one or more of the known carriers that transport similar organic anions and zwitterions [29]. There are 11 known carriers in the mitochondrial inner membrane of cells such as hepatocytes or renal PT cells that transport organic anions and zwitterions such as citric acid cycle intermediates, amino acids, and gluconeogenic precursors. Each of these carriers is energized by counter-transport with other ions and is either electroneutral (*i.e.*, no net transfer of charge across the mitochondrial inner membrane) or electrogenic (*i.e.*, net transfer of charge across the mitochondrial inner membrane). These include three adenine nucleotide translocases (AAC1-3; *Slc25a4-6*; exchanges ATP for ADP and is electrogenic), the phosphate carrier (PiC; *Slc25a3*; exchanges phosphate for hydroxyl ions and is electroneutral), the dicarboxylate carrier (DIC; *Slc25a10*; exchanges dicarboxylates such as malate for phosphate and is electroneutral), the 2-oxoglutarate carrier (OGC; *Slc25a11*; exchanges 2-oxoglutarate for other dicarboxylates such as malate and is electroneutral), two glutamate-aspartate carriers (AGC1/2; *Slc25a12/13*; exchanges glutamate and a proton for aspartate and is electrogenic), two glutamate-hydroxide ion carriers (GC1/2; *Slc25a22/18*; exchanges glutamate for hydroxyl ions and is electroneutral), the tricarboxylate or citrate carrier (CIC; *Slc25a1*; exchanges citrate and a proton for dicarboxylates such as malate and is electroneutral), and the monocarboxylate carrier (MCC; exchanges monocarboxylates such as pyruvate and lactate for hydroxyl ions and is electroneutral).

**Figure 2:**
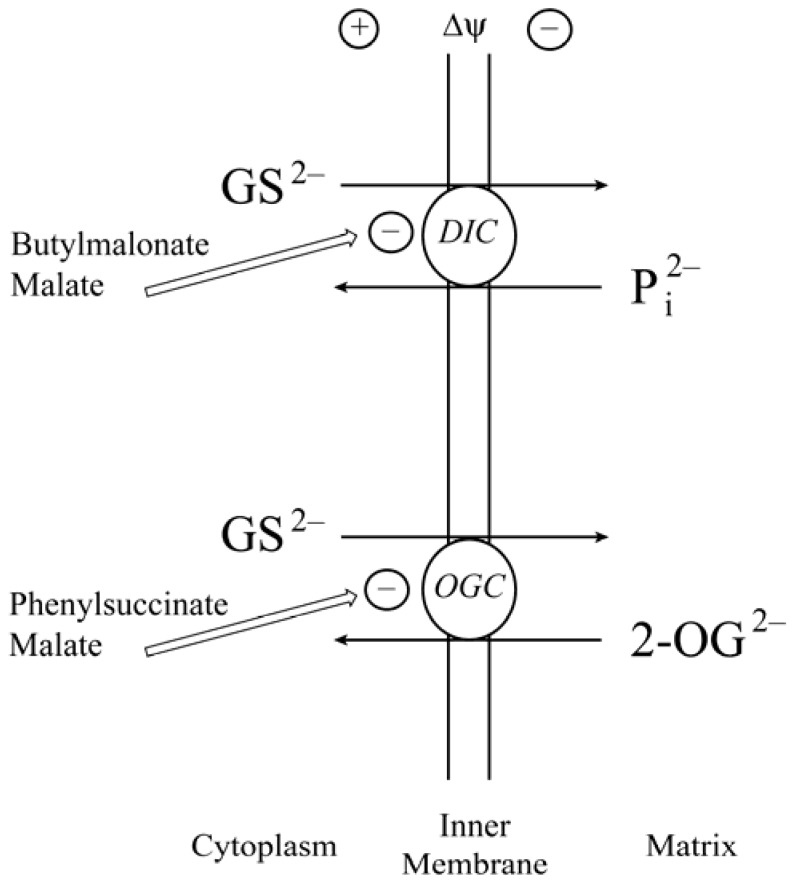
Pathways of glutathione transport across the mitochondrial inner membrane. Minus signs after each arrow indicate inhibition of the transporter activity. Abbreviations: DIC, dicarboxylate carrier; GS^2^^−^, glutathione; OGC, 2-oxoglutarate carrier; P_i_^2^^−^, inorganic phosphate.

Our approach to identify potential mtGSH carriers began with studies in suspensions of freshly isolated renal cortical mitochondria from rats to assess membrane potential dependence (*i.e.*, Yes = electrogenic transport; No = electroneutral transport), substrate specificity, dependence on inorganic phosphate concentration, and sensitivity to selective inhibitors of specific carriers [28,30]. GSH uptake in isolated renal cortical mitochondria from rats was not dependent on membrane potential, was significantly inhibited in a nominally phosphate-free incubation medium, and was competitively inhibited by *S*-alkyl derivatives of GSH, γ-glutamyl amino acids, and several dicarboxylates but not by monocarboxylates. Further, transport was also inhibited by the DIC- and OGC-selective inhibitors butylmalonate and phenylsuccinate, respectively [30]. Hence, as illustrated in Figure 2, we concluded that of the 11 organic anion/citric acid cycle intermediate carriers on the mitochondrial inner membrane, two of them, the DIC and OGC, are capable of catalyzing GSH transport in renal cortical mitochondria.

Function of the DIC and OGC in the transport of cytoplasmic GSH into the mitochondrial matrix was further demonstrated in studies in which inner membrane carriers were partially purified from rabbit kidney cortex and reconstituted into proteoliposomes [31]. This methodology has the advantage of enabling study of transport without the potential confounding of intracellular or intraorganellar metabolism. Kinetics, substrate specificity, energetics, and sensitivity to butylmalonate and phenylsuccinate in the reconstituted system were all similar to those observed in intact mitochondria.

Experiments in isolated mitochondria or reconstituted proteoliposomes are valuable to characterize mechanisms of transport. They are limited, however, in that the relationship between transport and cellular function cannot be studied. To address this limitation, we used an immortalized cell line derived from Normal Rat Kidney Epithelial cells called NRK-52E cells [32]. This cell line has the advantage of possessing many properties and functions of normal rat renal PT cells, including a relatively high density of mitochondria. Despite the high density, expression of mitochondrial inner membrane anion carriers such as the DIC and OGC are very low in NRK-52E cells. This enables expression of a desired amount of carrier proteins to further study their roles in cellular function and chemical toxicity [33,34]. Some additional aspects of mtGSH transporter manipulation in NRK-52E cells will be discussed in Section 4.

### 2.2. Function of the DIC and OGC Demonstrated in Mitochondria from Other Tissues

Although the focus of this review is on mtGSH in diabetic kidneys, a brief discussion of the evidence for mtGSH in other tissues and from other research groups is warranted. Despite some degree of heterogeneity, the DIC and OGC are present in all mitochondria [22,35]. Consistent with this fact, we demonstrated function of the DIC and OGC in mtGSH transport in isolated rat liver mitochondria and H4IIE rat hepatoma cells [36]. Competition experiments in isolated rat liver mitochondria with other potential transport substrates, sensitivity to butylmalonate and phenylsuccinate, and energetics showed that the DIC and OGC can account for most, although not all of the transport of GSH into hepatic mitochondria. Stable overexpression of the cDNA for the OGC in H4IIE cells provided further evidence by showing a marked increase in uptake of both GSH and 2-OG and protection of cells from acute cytotoxicity caused by either hydrogen peroxide, methyl vinyl ketone, or cisplatin. Additionally, Kaplowitz and colleagues [37,38] provided direct evidence for the function of the OGC in hepatic mtGSH transport by showing that overexpression of the OGC from HepG2 cells in Xenopus laevis oocyctes resulted in GSH transport that was inhibited by phenylsuccinate. They further demonstrated that transport of GSH and 2-OG in isolated rat liver mitochondria was competitive with each other and both were inhibited by phenylsuccinate.

Function of the DIC and/or OGC in mtGSH transport has also been clearly and definitively shown by studies in brain tissue [39,40,41] and colonic epithelial cells [42,43]. In rat brain mitochondria, mtGSH transport was inhibited by butylmalonate but not by phenylsuccinate, suggesting that it is the DIC rather than the OGC that is mainly responsible for the transport. Knockdown of DIC expression with short-hairpin RNA in neuronal-like PC-12 cells resulted in markedly decreased transport of GSH into mitochondria and increased ROS content, primarily in the form of hydrogen peroxide. Analysis of primary cultures of rat cerebellar granule neurons and cerebellar astrocytes showed that mitochondria from the former only expressed the DIC whereas both OGC and DIC proteins were expressed in the latter cells [41]. Consistent with this expression pattern, butylmalonate specifically reduced mtGSH content in cerebellar granule neurons whereas both butylmalonate and phenylsuccinate were able to reduce mtGSH content in astrocytes. Knockdown of DIC expression with adenoviral siRNA increased susceptibility of cerebellar granule neurons to oxidative stress. Similar types of studies in an immortalized and non-malignant human colonic epithelial cell line, NCM460 cells [42,43], also showed a direct relationship between expression and activity of DIC or OGC, mtGSH concentrations, and susceptibility to oxidative injury induced by menadione.

Thus, although other as yet unidentified carriers may participate in mtGSH transport to varying degrees depending on the tissue, the DIC and OGC are the major carriers for transport of GSH from cytoplasm to mitochondrial matrix in all tissues examined thus far, which includes, kidney, liver, brain, and colon.

### 2.3. Do the DIC and OGC Really Transport GSH into Mitochondria?

Despite the wealth of data from multiple laboratories and studies in multiple tissues, cell lines, and subcellular fractions from multiple tissues, Booty and colleagues recently published a paper in *FEBS Letters* [44] concluding that neither the DIC nor the OGC from yeast and human, respectively, can transport GSH. These authors studied dicarboxylate and GSH transport in fused membrane vesicles of *Lactococcus lactis* that overexpressed the two carriers. The cDNA for the DIC was from yeast because the protein expressed using the human cDNA exhibited negligible function. They found no evidence of GSH being transported by these reconstituted carriers or for GSH interacting with the readily measurable transport of malate. The conclusion these authors came to was that “these mitochondrial carriers do not transport GSH and the identity of the mitochondrial GSH transporter remains unknown”. The paper makes four incorrect, unsubstantiated and/or misleading assertions.

*Assertion #1:* The rationale used by these authors for their study is that the measured mtGSH transport activity in the various systems used by multiple laboratories was “poorly characterized”. It seems rather odd and inappropriate to categorize a body of work that has examined kinetics, energetics, substrate specificity, dependence on membrane potential, and sensitivity to several carrier-selective inhibitors as “poor characterization”.

*Assertion #2:* The authors then claim to use the “well-established *Lactococcus lactis* system for overexpression and characterization of members of the mitochondrial carrier family”. The references cited to support use of this experimental system examined yeast carriers, which are quite different from mammalian carrier proteins. They further denigrate use of *E. coli* overexpression that we [31] and others [45,46,47,48,49] had used, yet fail to demonstrate any clear advantage to the system they used.

*Assertion #3:* The transport of GSH by reconstituted inner membrane carriers is concluded to not exhibit typical properties of mitochondrial carriers because the transport ceased after 2 min. This is an incorrect conclusion as transport in proteoliposomes is limited due to rapid equilibration of any concentration gradients. Similar to what is typically observed with membrane vesicles, accumulation of transported substrate in proteoliposomes reaches a maximum and then decreases to reach an equilibrium level as the energetic driving forces for transport are dissipated. A time course thus typically shows an over-shoot.

*Assertion #4:* The authors cite cellular effects of overexpression of the DIC and OGC in our previous studies [33,34,36] as leading to “a number of interesting effects … that are likely to be secondary consequences of metabolite redistribution between the mitochondria and cytosol, and cannot be taken as evidence of changes in GSH transport”. While it is certainly true that it is difficult to separate effects due to a specific transporter from those due to metabolism or redistribution of other metabolites in complex systems such as intact cells, the goal in those studies was to examine the impact of different levels of transporter function on integrated cellular processes (e.g., chemically induced apoptosis). These effects were associated with expected changes in mtGSH concentrations and were prevented by application of the selective DIC and OGC inhibitors butylmalonate and phenylsuccinate, respectively. Hence, although the impact of secondary effects certainly cannot be excluded, to conclude that only such secondary effects and not transport is responsible is premature and not supported by the entire body of data.

Hence, in response to the question posed as the title of this subsection, the answer is: Yes, the DIC and OGC do transport GSH from the cytoplasm into the mitochondrial matrix in renal PT cells, hepatocytes, and other mitochondria that have been studied thus far. Known properties of these carriers, such as substrate competition, electroneutrality, stimulation of GSH uptake by preloading with counter substrates such as malate or inorganic phosphate, inhibition by butylmalonate and phenylsuccinate, and enhancement of uptake and mitochondrial accumulation of GSH in cell lines in which either the cDNA of the DIC or OGC have been overexpressed. While the participation of additional carriers, particularly in hepatocytes [36], cannot be excluded, the data from multiple laboratories in multiple experimental models support the function of the DIC and OGC as the major mitochondrial inner membrane carriers for the movement of GSH from the cytoplasm into the mitochondrial matrix. It should also be noted that the cDNA for the yeast DIC only has 40% homology with that of humans or rats. Hence, transport properties may differ significantly across species.

## 3. Cellular and mtGSH in Diabetic Nephropathy and Other Renal Diseases Involving Tubular Epithelial Cell Hypertrophy

### 3.1. Diabetic Nephropathy

As noted above, changes in mitochondrial function are widely recognized to play a fundamental, underlying role in the biochemical and molecular changes in target tissues such as the kidneys that are adversely affected by diabetes. Depletion or oxidation of renal cellular GSH has been observed in several models of diabetic nephropathy [13,14,15,16,17,18]. To systematically assess the impact of diabetic nephropathy on renal PT cell function and GSH status, we produced diabetes in male Sprague-Dawley rats with streptozotocin (STZ) and examined renal function, mitochondrial function, and the status of GSH and other antioxidants at 1 month and 3 months after induction of diabetes [50]. STZ generates a Type 1 diabetes and nephropathy is a well-established pathological effect that typically occurs within 45 to 60 days after injection and establishment of hyperglycemia [52,53]. Hence, we chose 1 month and 3 months as time points to examine both early effects prior to the onset of frank nephropathy and those effects after nephropathy was clearly established.

Surprisingly, significant changes in renal function in 1-month diabetic rats were observed, including lower body weight gain and increased kidney weight, plasma *N*-acetyl-β-d-glucosaminidase (NAG), urinary albumin, and urinary protein as compared to control rats [50]. Moreover, hematoxyl and eosin (H&E) and *p*-amino Shiff base (PAS) staining indicated modest development of proximal tubular hypertrophy, thickening of the glomerular basement membrane, glomerular hypertrophy, and mesangial expansion. State 3 respiration in suspensions of isolated renal cortical mitochondria from diabetic rats was also significantly higher than in those from 1-month control rats. Thus, adaptations to the chronic hyperglycemic state were already apparent at this early stage of diabetes. Renal damage, as indicated by plasma NAG, urinary albumin and protein, and histopathology, were exacerbated by 3 months of diabetes, indicating a progression of DN.

**Figure 3:**
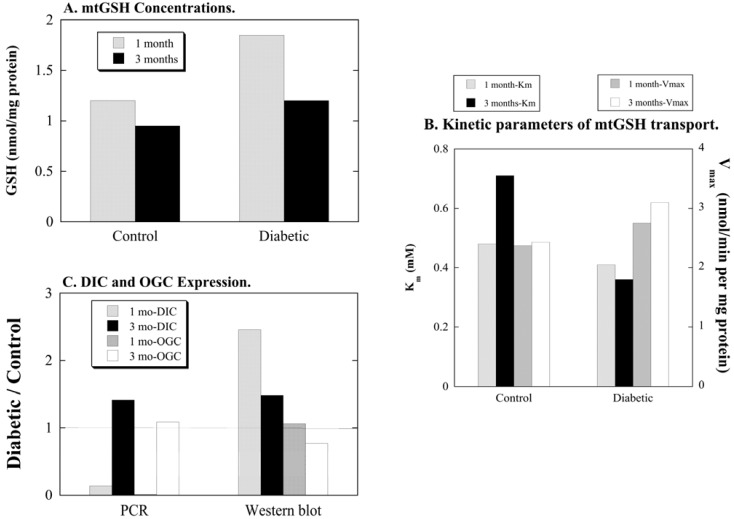
Summary of selected data on mitochondrial glutathione status in kidneys of diabetic rats. Data are summarized from a previously published study [50] and are shown without error bars for simplicity. Values are means of measurements from 4 control and 4 diabetic rats for panel (**A**) and (**B**). For panel (**C**), PCR values are means of measurements from 9 and 12 control and 9 and 12 diabetic rats for 1-month and 3-month studies, respectively; Western blot values are means of measurements from 3 control and 3 diabetic rats. (**A**) Mitochondrial GSH (mtGSH) concentrations were determined with the GSH-Glo^TM^ chemiluminescence kit from Promega (Madison, WI, USA). (**B**) mtGSH transport into isolated mitochondria was measured with ^35^S-labeled GSH as described in reference [50]. (**C**) Real-time quantitative PCR measurements of DIC and OGC mRNA in renal cortical samples were done as described in reference [50], using glyceraldehyde 3-phosphate dehydrogenase (GAPDH) as an endogenous control and the ∆C_T_ method for determination of relative expression levels. (**D**) Western blots were performed by enhanced chemiluminescence with rabbit anti-rat polyclonal antibodies to the DIC and OGC. Expression levels were normalized to that of the voltage-dependent anion channel (VDAC) and band density was derived using GelEval 1.3.7 software for Mac OS X.

In contrast to the progressive changes in renal damage, several aspects of GSH status exhibited early changes at 1 month that partially normalized by 3 months, suggesting compensation and adaptation (Figure 3). Specifically, both cytoplasmic and mitochondrial GSH concentrations in renal cortex were about 50% higher in 1-month diabetic rats than in corresponding fractions of 1-month control rats; at 3 months, however, cytoplasmic and mitochondrial GSH concentrations in renal cortex were only 20% to 30% higher in diabetic rats than in corresponding fractions of control rats. GSH synthesis activities, as measured by activity of glutamate-cysteine ligase, were similar in kidneys of diabetic and control rats at 1 month and were about 35% higher in kidneys of diabetic rats at 3 months. GSH peroxidase (GPX) activity was about 40% and 100% higher in kidneys of diabetic rats than in those of control rats at 1 month and 3 months, respectively, suggesting a chronic state of oxidative stress. While K_m_ values for mtGSH transport did not markedly differ between renal mitochondria of control and diabetic rats, V_max_ values were significantly higher by approximately 15% and 30% in renal mitochondria of diabetic rats at both 1 month and 3 months, respectively. Although mRNA expression of the DIC and OGC did not follow the same patterns, protein expression of the DIC but not OGC was higher in renal mitochondria from diabetic rats.

In addition to mtGSH status, other antioxidants in renal mitochondria were also assessed. Protein expression of superoxide dismutase 2 (SOD2) was modestly higher in renal mitochondria from diabetic rats at both 1 month and 3 months whereas that of thioredoxin 2 (Trx2) did not differ between kidneys of control and diabetic rats. To further assess oxidative stress, two types of measurements were made to detect oxidatively modified proteins and lipid peroxidation in isolated renal mitochondria. Two assays of oxidatively modified proteins were conducted, Western blots of 4-hydroxy-2-nonenal (HNE)- and 3-nitrotyrosine (3-NT)-adducted proteins. While no differences were observed in levels of 3-NT-adducted proteins, significant differences in patterns of HNE-adducted proteins both between renal mitochondria of control and diabetic rats and between samples at 1 month and 3 months. Measurements of both basal and oxidant-stimulated malondialdehyde (MDA) formation were similar in corresponding samples from renal mitochondria of control and diabetic rats at both time points. These results suggest that whereas RNS are not likely involved, differences in levels of ROS do contribute to some extent to changes in redox status in DN.

Besides impaired renal function, we hypothesized that another impact of DN is an altered susceptibility of renal PT cells to chemically induced injury [51]. To test this, we characterized baseline redox status and compared responses of primary cultures of renal PT cells from control and 1-month, STZ-treated diabetic rats to two oxidants (tert-butyl hydroperoxide [tBH] and methyl vinyl ketone (MVK)) and a mitochondrial toxicant (antimycin A (AA)). Untreated renal PT cells from diabetic rats were clearly hypertrophied as compared to those from control rats. Untreated renal PT cells from diabetic rats also exhibited higher baseline fluorescence when incubated with 2,7-dichlorofluoroscein (DCFH), suggesting higher basal levels of hydrogen peroxide or peroxynitrite [54]. In contrast, no differences were observed in fluorescence of cells incubated with dihydroethidium, which suggests that basal levels of superoxide anion do not differ. Finally, untreated renal PT cells from diabetic rats exhibited markedly higher baseline fluorescence when incubated with 5,5′,6,6′-tetrachloro-1,1′,3,3′-tetraethylbenzoimidazolyl carbocyanine iodide (JC-1), indicating higher mitochondrial membrane potential. These findings in primary cultures of renal PT cells agree with those from the *in vivo* studies, showing that kidneys from diabetic rats, in particular the PT cells, are hypertrophied, exhibit an elevated basal of ROS, and have hyperpolarized mitochondria. Challenge of renal PT cells with tBH, MVK, or AA revealed that the cells from diabetic rats were generally more susceptible to injury, particularly from the mitochondrial toxicant AA. This suggests that the existence of DN produces an added risk for drug and chemically induced renal injury and should be an important consideration in human health risk assessment.

### 3.2. Uninephrectomy and Compensatory Renal Hypertrophy

As noted above, one of the characteristics of renal PT cells from DN rats is cellular hypertrophy. Another model of renal PT cellular hypertrophy that we have studied with respect to GSH status and mitochondrial function is that of uninephrectomy and compensatory renal growth [55,56,57,58]. Reductions in functional renal mass, which are associated with aging and several renal diseases, result in a compensatory response in the remnant renal tissue that is primarily a hypertrophy rather than a hyperplasia response. While some of the effects of compensatory renal hypertrophy (CRH) on cellular metabolism and redox status differ from that due to the cellular hypertrophy associated with DN, many are the same or very similar. Thus, studies on changes in mitochondrial function and redox status in CRH can provide some insight into events that occur in mitochondria of renal PT cells of rats with DN.

Rats that underwent uninephrectomy (NPX rats) exhibited elevated kidney weights, increased serum creatinine, and increased levels of several urinary markers of kidney function, including protein, albumin, NAG, and γ-glutamyltransferase (GGT) [59], suggestive of mild kidney damage due to compensatory hypertrophy. Similar to the hypertrophied PT cell in diabetic rats, the hypertrophied PT cell from NPX rats exhibited increased mitochondrial respiration and increased rates of mtGSH uptake. Despite the increased rates of mitochondrial respiration and higher levels of basal ROS, mtGSH concentrations were significantly higher in renal cortical mitochondria of NPX rats as compared to those of control rats [55,56,57,59]. Despite the higher rate of mtGSH uptake in these mitochondria, no differences were observed in either mRNA or protein expression of the two carriers. It was concluded that the apparent compensatory increase in mtGSH transport activity was due to the hypermetabolic state of renal mitochondria due to increased fluxes of intermediary metabolites, including various dicarboxylates or their precursors that are substrates for the DIC and/or OGC. Thus, whereas the compensatory increase in mtGSH in renal PT mitochondria from diabetic rats is in part due to increased expression of DIC protein, that in renal PT mitochondria from NPX rats appears to be due exclusively to kinetics or mass action effects.

**Figure 4:**
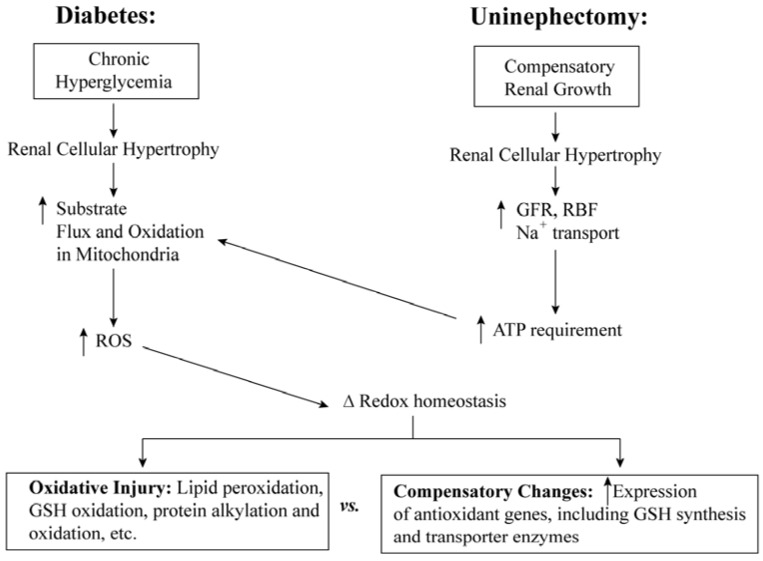
Scheme of functional and biochemical responses to diabetes or uninephrectomy that are associated with changes in mitochondrial redox status. Abbreviations: GFR, glomerular filtration rate; GSH, glutathione; RBF, renal blood flow; ROS, reactive oxygen species. The scheme is based on one published in reference [25] with some modifications.

A schematic comparison on the effects on mitochondrial function and redox status of renal cellular hypertrophy arising from DN and uninephrectomy is shown in Figure 4. Although the initial cause (*i.e.*, chronic hyperglycemia *vs.* reduced functional nephron mass) differs, both events result in cellular hypertrophy and a hypermetabolic state. In the case of DN, the excess glucose results in increased flux of substrates to the mitochondria. In the case of uninephrectomy, the reduced functional nephron mass results in increased renal blood flow and ion transport, resulting in increased need for ATP and, therefore, increased mitochondrial activity. Ultimately, both models can exhibit compensatory changes in antioxidants such as GSH, but can progress to oxidative damage, particularly to the mitochondria.

A schematic comparison on the effects on mitochondrial function and redox status of renal cellular hypertrophy arising from DN and uninephrectomy is shown in Figure 4. Although the initial cause (*i.e.*, chronic hyperglycemia *vs.* reduced functional nephron mass) differs, both events result in cellular hypertrophy and a hypermetabolic state. In the case of DN, the excess glucose results in increased flux of substrates to the mitochondria. In the case of uninephrectomy, the reduced functional nephron mass results in increased renal blood flow and ion transport, resulting in increased need for ATP and, therefore, increased mitochondrial activity. Ultimately, both models can exhibit compensatory changes in antioxidants such as GSH, but can progress to oxidative damage, particularly to the mitochondria.

## 4. Toxicological Impact of Varying mtGSH Transport

With the presumption that changes in mitochondrial function and redox status underlie DN and many other diseases or pathological conditions, it would then seem to be a logical therapeutic strategy to target systems that can support maintenance of mitochondrial redox homeostasis. Further, the central role of mtGSH in this process would then make it a likely target for experimental manipulation. A major complication with this strategy, however, lies in the tight regulation and existence of feedback mechanisms involving both GSH synthesis in the cytoplasm and GST transport into the mitochondria that limit the ability to significantly alter mtGSH concentrations in a sustained manner. Typically, efforts to increase concentrations of an antioxidant, including GSH, result in only transient increases and eventual re-equilibration back to previous concentrations. Any strategy that hopes for an effective and sustained response must involve changing the set point for the antioxidant so that re-equilibration does not occur or that the equilibration point is permanently changed. To reiterate the key point, just incubating cells or tissues with the antioxidant of interest (GSH in this case), may provide a transient improvement in cellular or mitochondrial redox status and a modest but short-term protection from chemical induced injury. In the long-term, however, a more sustained effect if needed.

As a proof of principle for the concept that modulation of the expression of mtGSH transporters can produce a sustained change in mtGSH concentrations and alter the susceptibility of the cell to oxidants and mitochondrial toxicants, we overexpressed the cDNA for rat DIC [33] or OGC [34] in NRK-52E cells. In the studies on the DIC, NRK-52E cells were transiently transfected with the rat cDNA for the DIC, resulting in a 2.45-fold and 11.3-fold increase in rates of transport of GSH and malonate, respectively, as compared to non-transfected cells. The transfected cells exhibited markedly less apoptosis than non-transfected cells from the oxidant tBH or the mitochondrial nephrotoxicant *S*-(1,2-dichlorovinyl)-l-cysteine (DCVC). In the studies on the OGC, NRK-52E cells were stably transfected with the rat cDNA for the OGC and exhibited 10- to 20-fold higher mtGSH transport activity than non-transfected cells. As with the transient transfection with the DIC cDNA, stable overexpression of the OGC resulted in a marked reduction of tBH- and DCVC-induced apoptosis as compared to non-transfected cells.

To provide further validation of the principle that modulation of mtGSH concentrations by manipulation of transporter expression can alter susceptibility to toxicants, NRK-52E cells were also stably transfected with a rat cDNA for a double-cysteine mutant OGC (rOGC-C221,224S) [34]. The rat OGC contains two cysteinyl residues at positions 221 and 224 that form an intramolecular disulfide bond during protein maturation [47]. Using site-directed mutagenesis, the two cysteinyl residues were converted to serines, thereby eliminating the disulfide linkage. Expression of the mutant carrier in NRK-52E cells resulted in an 80% decrease in catalytic efficiency as compared to the wild-type OGC. While accumulation of mtGSH was only modestly lower than that of non-transfected cells, NRK-52E cells expressing the rOGC-C221, 224S mutant protein were equally sensitive to tBH- and DCVC-induced apoptosis as the non-transfected cells.

Such an experimental approach has not been conducted yet in primary cultures of renal PT cells from diabetic rats. However, these cells were markedly protected from toxicity from either the oxidants tBH or MVK or the mitochondrial toxicant AA by treatment with *N*-acetyl-l-cysteine (NAC) [51], which can act as both a direct antioxidant and as a precursor for GSH. NAC co-treatment of toxicant-treated cells resulted in decreased ROS formation, decreased proportion green fluorescence from JC-1, indicating improvement in mitochondrial membrane potential, and decreased LDH release, apoptosis, and evidence of morphological damage.

Preliminary studies to assess the impact of overexpression of the DIC or OGC in primary cultures of renal PT cells from NPX rats have, however, been conducted and support this as a potential therapeutic strategy [60]. As discussed above, renal PT cells from NPX rats, like those from DN rats, are hypertrophied and exhibit a hypermetabolic state and an elevated level of mitochondrial oxidative stress. Although certain steps in the pathways by which these effects arise differ between the two cellular hypertrophy models, studies with renal PT cells from NPX rats can still be informative for responses in renal PT cells from DN rats. Another similarity between hypertrophied renal PT cells from NPX and DN rats is that the former are also more susceptible than cells from normal rats to various oxidants and other toxicants [61,62].

Overexpression of rat cDNA for either the DIC or OGC in primary cultures of renal PT cells from NPX rats resulted in protection from AA- but not tBH- or MVK-induced cytotoxicity. Although protein levels of the two transporters were not measured, measurement of mRNA levels showed a several thousand-fold increase for each carrier. Additional studies are needed to quantify the functional changes in transport after cDNA overexpression and to correlate them with mtGSH levels. Further, a method of transfection that enables a more tightly regulated level of expression needs to be optimized for the primary cultures. Nonetheless, the clear protection against a mitochondrial toxicant supports the overall experimental approach.

## 5. Summary and Conclusions

DN is a significant public health concern that affects an increasing number of individuals. Little effective treatment is available and as the disease progresses to end-stage renal disease, renal replacement therapy (*i.e.*, dialysis) and eventually organ transplantation are the only options. Accordingly, there is great need for development of novel and effective therapeutic approaches. DN is at its foundation a metabolic disease, with mitochondrial dysfunction being a critical, underlying factor in its development. Moreover, development of mitochondrial dysfunction is closely associated with oxidative stress, in particular involving alterations in GSH status. Based on these points, we hypothesized that modulation of the systems that determine the status of mtGSH may be good targets for development of therapeutic approaches to treat DN.

Studies in isolated mitochondria from rat kidney cortex, primary cultures of rat renal PT cells, a stable cell line derived from normal rat kidney (*i.e.*, NRK-52E cells), and with a partially purified and reconstituted preparation of mitochondrial inner membrane transporters identified the DIC and OGC as the principle membrane carriers that mediate the transport of GSH from the cytoplasm into the mitochondrial matrix. Inasmuch as GSH synthesis appears to occur exclusively in the cytoplasm, function of these membrane carriers provides the mtGSH pool. Other studies from multiple laboratories in cells or subcellular fractions derived from rat liver, rat brain, and rat colonic epithelial cells also showed that these two carriers play significant roles in mtGSH transport.

Having identified the primary carriers for GSH in the mitochondrial inner membrane thereby provided specific molecular targets for development of therapeutic approaches to enhance mtGSH and improve the bioenergetics function and redox state of renal mitochondria from DN rats. Preliminary studies in primary cultures of renal PT cells from DN rats and in those from NPX rats provided experimental support for the concept that overexpression of either the DIC or the OGC can be a viable strategy to achieve a positive therapeutic outcome.

The importance of the DIC and OGC and other mitochondrial inner membrane carriers is highlighted by a review by Palmieri [63] that highlights human diseases associated with genetic defects in these carriers. Moreover, a special issue published in 2013 in *Molecular Aspects of* *Medicine* devoted to the pharmacological, toxicological, and pathological implications of transporter function further emphasizes the growing appreciation of how membrane transporters can be targets for drug development or how defects in their function can be the basis of disease. Three papers from this issue included one that reviewed solute carriers as drug targets [64], one that reviewed the role of membrane transporters in drug-drug interactions and their influence on regulatory decisions for new drug approval [65], and a review on the role of solute carriers as targets for treatment of cancer [66].

**Figure 5:**
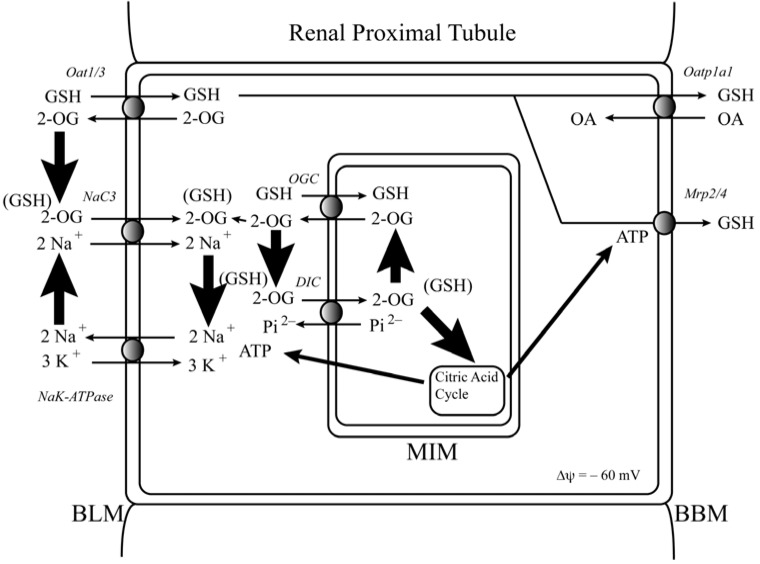
Pathways of glutathione transport in renal proximal tubular cells. Shown are the major pathways for glutathione (GSH) across both plasma membranes and the mitochondrial inner membrane (MIM). The critical importance of mitochondrial energetics in determining both mitochondrial and overall cellular GSH status is apparent from the integration of transport pathways with the citric acid cycle and ATP generation. The figure is based on one published in reference [25] with slight modifications. Abbreviations: BBM, brush-border membrane; BLM, basolateral membrane; DIC, dicarboxylate carrier; Mrp2/4, multidrug resistance proteins 2/4; NaC3, sodium-dicarboxylate carrier 3; OA, organic anion; Oat1/3, organic anion transporter 1/3; Oatp1a1; organic anion transporting polypeptide 1a1; 2-OG, 2-oxoglutarate; OGC, oxoglutarate carrier; P_i_^2^^−^, inorganic phosphate.

The integration and coupling of membrane transport processes for GSH with mitochondrial function and intermediary metabolism in the renal proximal tubule is highlighted in Figure 5. Besides GSH synthesis requiring ATP, GSH transport across both the mitochondrial inner membrane and the basolateral plasma membrane are in part coupled to gradients of 2-OG. Transmembrane gradients of 2-OG are in turn generated by citric acid cycle activity in mitochondria and function of a secondary active transporter (the sodium-dicarboxylate 3 (NaC3; *Slc13a3*)) and a primary active transporter (the Na^+^+K^+^-stimulated ATPase) on the basolateral plasma membrane. Hence, it is clear that these various transport systems are highly integrated. Although the complexity of these processes may produce unexpected effects when one or more components are manipulated, identification of the various components provides unique opportunities for future work on development of novel therapeutic approaches to DN as well as other diseases for which mitochondrial dysfunction and oxidative stress are underlying features.
